# The impact of patentees assessment based on the heterogeneous patent innovation network

**DOI:** 10.1016/j.heliyon.2024.e30317

**Published:** 2024-04-27

**Authors:** Xipeng Liu, Xinmiao Li

**Affiliations:** a School of Management, Shanghai University, Shanghai, 200444, China; b School of Information Management and Engineering, Shanghai University of Finance and Economics, Shanghai, 200433, China

**Keywords:** Patentee impact, Heterogeneous patent innovation network, *CWAPN*, Chinese green patents, Energy field

## Abstract

As a vital factor in technological innovation, patentee plays a significant role in the process of scientific and technological innovation, researching patentee has attracted the attention of experts and scholars. Previously, scholars have mainly quantified patent indicators or constructed homogeneous information networks to analyze patentees, but these methods cannot objectively measure the impact of patentees. Therefore, this study proposes a novel approach to assessing patentee impact based on a heterogeneous information network. The proposed method distinguishes the weight of different types of nodes using a weighted mechanism and extracts three types of fine-grained characteristics of network nodes. This approach results in the construction of a heterogeneous patent innovation network and the development of a new patentee impact assessment algorithm called *CWAPN*. Using Chinese green patents in the field of energy conservation and environmental protection as an example, experimental results show that the *CWAPN* algorithm can effectively assess the impact of patentees. Thereby identifying patentees who have made outstanding contributions to sustainable development in China.

## 1 Introduction

Patent as the result of innovation, it records the results and trajectory of invention, and patentee is the subject of patent rights. As the most active factor in patent activity, patentee plays a crucial role in the process of scientific and technological innovation. Consequently, analyzing patent quality and patentee impact can help identify important innovation subjects, core patent compositions, and competition and cooperation among technology subjects. Such analyses also provide objective data for science and technology management, policy formulation, and technology strategic layout decision. In previous studies, scholars evaluated the impact and innovation ability of patentees based on the number of patents or citations. The more patents a patentee invents, the greater their impact; and the more citations a patent receives, the stronger the patentee's innovation ability [1,2]. In addition, patentometrics has borrowed from bibliometrics analysis methods such as the H index [3] and G index [4] to evaluate the impact of patentees. However, these methods excessively rely on the number of patent citations and can result in significant bias in patentee rankings, and it hinders the objective assessment of the impact of patentees.

To address the limitations of using patent and citation data to assess the impact of patentees, researchers have turned to building network structures. Patent data typically includes patent number, patentees, inventors, citation relationships, and patentee affiliation relationships. In previous studies, most scholars have utilized homogeneous information networks to analyze the impact of patentees. For instance, constructing citation networks of patentees based to their citations and affiliations [5]. Alternatively, it can construct a citation network for patents to calculate the importance score of patents before calculating the impact of patentees based on patent affiliation [6], or constructed collaborative networks to analyze influential patentees in technology based on patentee partnerships [7]. However, “homogeneous information networks” refer to those networks that only contain one type of node and one type of relationship, which may ignore the impact of other types of entities and relationships in the dataset. In contrast, “heterogeneous information networks” encompass a broader range of information, incorporating diverse types of entities and various relationships among them. As a result, it has found extensive applications in analyzing complex social relationships between different entities [8,9]. Heterogeneous information networks span various domains, including academic information networks, social media networks, and medical information networks, etc. Despite this, there has been limited research on patent innovation networks. Unlike homogeneous patent innovation networks that only incorporate homogeneous information networks related to individual patents, we have developed a heterogeneous patent innovation network encompassing two distinct entities: patents and patentees. To provide a more objective assessment of the impact of patentees, we have developed a heterogeneous patent innovation network using principles from complex network theory. This network efficiently mitigates the problems of self-citation encountered in previous homogeneous patent networks, as well as the mutual citation issues in patent citation networks. Furthermore, we utilized a weighted mechanism to differentiate the importance of nodes belonging to different categories and identify three crucial characteristics within the network's nodes. This approach results in a more sensible evaluation of the impact of patentees. Therefore, this study aims to construct a heterogeneous patent innovation network and employ the widely used PageRank algorithm to calculate the impact of entities in the network. While the PageRank algorithm provides a recursive method for analyzing the global structure of networks [10], heterogeneous information networks incorporate multiple types of entities and relationships, requiring a weighted mechanism to distinguish the importance of various entities. Moreover, node characteristics in networks play a significant role in evaluating impact. Therefore, this study attempts to explore node characteristics in the heterogeneous patent innovation network, including the credit allocation value of patentees, the H index of patentees, and the time decay factor of patents.

In the context of advocating green economic development, green technological innovations contribute to achieving the goals of enhancing enterprise competitiveness and promoting environmental protection for sustainable development. Green patents, as significant carriers of technological innovation, facilitate the analysis of these patents to unearth crucial innovators within this domain. This comprehensive understanding of our country's green innovation market conditions provides objective data support. Consequently, we collected and matched 539,297 green innovation patent records from the China National Intellectual Property Administration for the years 1985–2020. Using this dataset as our research target, we constructed a novel heterogeneous innovation network and evaluated the impact of patentees. The experimental results demonstrated that the *CWAPN* algorithm performs well.

The contributions of this study included the following three main parts.

(1) A novel approach to construct a heterogeneous patent innovation network is proposed, which is different from homogeneous information networks in previous studies. The constructed network contains rich structural and information, providing a more objective assessment approach for identifying influential patentees.
(2) In the proposed heterogeneous patent innovation network, a weighted mechanism is utilized to distinguish the weights of various types of nodes. Moreover, three categories of node characteristics are extracted to enhance the PageRank algorithm. The *CWAPN* algorithm not only considers the interplay between different entities in heterogeneous information networks, but also considers the impact of network structure and node characteristics on the significance of patentees.
(3) This algorithm not only provides a more accurate assessment of the impact of patentees, but also effectively addresses the recommendation problem in the patent innovation network. It is helpful for analyzing the position of patentees in the green innovation environment in China and providing decision-making support for companies or organizations to develop R&D strategies.

The subsequent sections of this paper are organized as follows. In Section 2, we present the literature review of this study. In Section 3, we describe the patentee assessment algorithm based on the heterogeneous patent innovation network. In Section 4, we introduce the data used in this study, baseline algorithms and relevant evaluation indicators. In Section 5, we show the results and analysis. Finally, in Section 6, we draw conclusions and future research prospects are discussed.

## 2 Literature review

In the context of patent innovation activities, patentees are the most active factor and play a crucial role in the innovation process. Consequently, evaluating the impact of patentees is not only an important research topic in the fields of patent analysis and technological innovation, but also holds significant practical implications for promoting technological innovation, managing technological risks, and enhancing technological competitiveness. In the existing literature, the algorithms designed for assessing the impact of patentees can be broadly categorized into two main categories: citation-based impact assessment algorithms and network structure-based impact assessment algorithms, these algorithms are summarized in Table 1.

**Table 1:** Summary of impact assessment algorithms.

Category	Reference	Core algorithm	Characteristics
Citation-based impact assessment algorithms	He et al. (2021)	Patent quantity	Proxy variable, easy and practical
	Hirsch (2005)	H index	Considering quantity of paper and citation count, age bias
	Egghe (2006)	G index	The enhanced H index considering the contributions of highly cited literature
Network structure-based impact assessment algorithms	Zheng et al. (2014)	AN_PRW	A patent collaborative network, belong to a homogeneous information network
	Miao et al. (2016)	PN_SPR	A patent citation network, ignore other entities and relationships.
	Kong et al. (2015)	TAPRW	A heterogeneous author-paper network
	Sayyadi et al. (2009)	FutRank	Considering network structure and time information
	Zhao et al. (2019)	APR	A heterogeneous author-paper network, ignore node's characteristics

### 2.1 Citation-based impact assessment algorithms

In previous studies, researchers have commonly used the number of patents as a proxy variable for innovation to measure the innovation capacity and impact of enterprises [11]. However, the quality of patents is also crucial in assessing the impact of patentees, as the innovative ability of a patentee is often demonstrated by the patents to which they belong. Therefore, the number of citations a patent receives is considered an important metric of its quality and market value, with more citations indicating higher degrees of innovation. Consequently, scholars have proposed a series of assessing algorithms that use citation-based algorithm to evaluate patent quality. One such algorithm is the H index, which was originally proposed to quantify scholars’ outcomes by synthesizing the quantity and quality of published papers [3]. Guan and Gao subsequently introduced the H index from bibliometrics to patentometrics, with experimental results demonstrating its effectiveness in assessing technological significance and impact [12]. Egghe proposed the G index to address the importance of highly cited literature that may be ignored by the H index [4]. Some scholars have used these assessment algorithms, such as the H index and G index, to calculate patentees and assess their patent impact from several dimensions [13,14]. All of these algorithms have successfully assessed patent impact and their computational processes are relatively simple and widely used.

However, when assessing the impact of patentees, relying solely on the number of citations for a patent can lead to biases for several reasons. Firstly, the number of citations for patents accumulates over time, and older patents have more time to accumulate citations compared to new inventions. Secondly, the frequency of patent citation in different technology fields may have different characteristics, and patentees in certain fields may be more inclined to cite the latest patent. To address these issues, researchers have proposed methods to weaken the influence of time or technological domain factors on innovation [15]. Furthermore, the number of citations for each patent takes time to accumulate and may not reflect the current innovative influence of the patentee [16]. Finally, some patentees attempt to increase the number of citations by various approaches, including self-citations or irrelevant citations from acquaintances such as friends, which can mislead patent impact scores and unfairly contribute to the patentee's innovation evaluation [17]. Therefore, it is necessary to develop more sophisticated algorithms to assess the impact of patentees, taking into account the aforementioned factors.

### 2.2 Network structure-based impact assessment algorithms

Several assessment algorithms based on the number of patent citations have been developed to evaluate the impact of patentees, but they didn't consider the quality of the citations. In general, if a patent is cited by a highly quality patent or authoritative innovator, it should carry more weight in assessing its importance. To address this issue, some scholars have proposed using network structure-based algorithms to assess the impact of patentees [[18], [19], [20]]. The core idea is that the importance of a node can be reflected by its position in the network, and if a node is at the core, it generally has a higher influence. Patent information datasets contain numerous entities and relationships, which are categorized into homogeneous and heterogeneous information networks based on the number of entities and relationships.

In constructing a homogeneous information network, only a single type of node and a single type of edge are considered. Therefore, when constructing a patentee information network, the patentee is the only node that exists, and depending on the patentee's affiliation, a cooperative network or a citation network can be constructed [7,21]. Some scholars have constructed patent citation networks to calculate patent impact scores and obtain the impact of relevant patentees. To analyze the impact of a patentee using network scientific methods, the position of the patentee in the network is analyzed, with greater influence being associated with closer proximity to the center. This assessment can be achieved by measuring indicators of network centrality, including degree centrality, betweenness centrality, closeness centrality, and eigenvector centrality. Additionally, common ranking algorithms such as PageRank and HITS are utilized to assess the impact of patentees. Taking the scholar impact assessment algorithms based on the network structure as an example [22], nodes represent scholars, while edges represent their collaboration relationships through co-authored papers. Scholars' influence in this academic collaboration network depends on their positions and connections within the network. The PageRank algorithm is employed to evaluate the “importance” of scholars in the academic network. A scholar is considered influential if they occupy a central position in the network and collaborate with other influential scholars. Using this algorithm, we can rank scholars based on their influence in the academic collaboration network and utilize these rankings to assess their academic reputation and contributions, thereby providing valuable information for academic decision-making.

Currently, most scholars analyzed patent data by constructing homogeneous information networks. However, patent datasets contain various types of entities, such as patent numbers, patentees, inventors, patent classification numbers, etc., and these entities are linked by different relationships, such as the reference relationship between patents, patent affiliation between patent and patentee, etc. Homogeneous information networks cannot fully present these information. In contrast, heterogeneous information networks can uncover different relationships between patent innovators and provide researchers with more comprehensive information [23]. Thus, the study of heterogeneous information networks has emerged in the field of patent innovation.

Tang et al. developed a universal theme-driven framework for analyzing and mining heterogeneous patent networks that comprise three object types: “companies, inventors, and technological content” [24]. Du et al. constructed inventor-patent heterogeneous networks based on patented inventors' co-inventive relationships and invention affiliations and proposed the InventorRanking algorithm, the results showed that the algorithm can effectively identify highly influential inventors [25]. Feng et al. effectively integrated patent features, such as patent documents, inventors, and applicants, to create a heterogeneous patent network with multidimensional features that can accurately identify technical innovation talent in patent datasets [26]. In the context of heterogeneous academic networks, Kong et al. modified the PageRank algorithm by incorporating the time of publication of different papers to assess the impact of scholars in the paper-scholar network of random walking algorithms [27]. Zhao et al. proposed the APR algorithm based on a heterogeneous author-paper network to assess the impact of scholars, defining the author's impact as the smooth distribution of the Markov process [22]. However, none of the aforementioned studies established a heterogeneous patent innovation network from the perspective of patentees, combined with patent citation relationships. Furthermore, the node features of the network contain essential values that can be mined and incorporated into the algorithm to enable random walking based on partial restarts, facilitating the identification of the most influential patentees in the field of patent innovation within heterogeneous information networks.

Therefore, we propose to construct a novel heterogeneous patent innovation network to assess the impact of patentees. This approach effectively avoids the severe bias issues caused by traditional quantified metrics such as H index or G index, and also mitigates the problem of self-citation or mutual citation in the constructed homogeneous information network, making the evaluation of patentee's influence more objective. Additionally, we not only take into account the mutual influence among different entities in the network but also consider the impact of network structure and node characteristics on the importance of patentees, resulting in more reasonable evaluation outcomes.

## 3 Patentee assessment algorithm based on the heterogeneous patent innovation network

In recent years, the explosive growth of patent information in China has provided a data basis for studying technological innovation. The evaluation of innovation impact is generally transformed into a statistical analysis or importance ranking problem. This study defines the impact of patentees as the probability of a patentee being randomly accessed in a heterogeneous patent innovation network. Firstly, a heterogeneous patent innovation network is constructed, and a weighted mechanism is used to distinguish the weight of different types of nodes. Additionally, the fine-grained information of network nodes must be considered, including the credit allocation value of patentees, the H index of patentees, and the time decay factor of patents. Finally, the PageRank algorithm is improved to obtain the impact assessment of patentees in the heterogeneous patent innovation network.

### 3.1 System framework

**Fig. 1:**
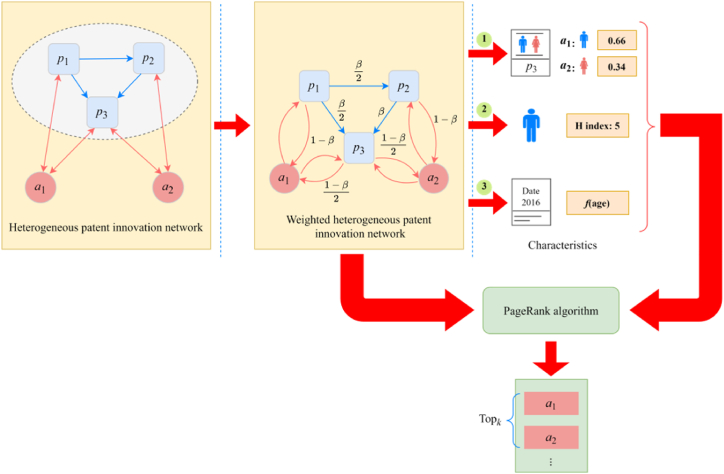
System framework.

This study proposes an algorithm for assessing the impact of patentees based on a heterogeneous patent innovation network. The system framework is shown in Fig. 1.

The system framework of the *CWAPN* algorithm is composed of four parts as follows.

(1) Constructing a heterogeneous patent innovation network with nodes consisting of patents and patentees. In this network, nodes $p_{1}$, $p_{2}$ and $p_{3}$ represent patents, while $a_{1}$ and $a_{2}$ represent patentees. There are two types of relationships in the network, represented by blue edges connecting patents, denoting citation relationships, and red edges connecting patentees, denoting patent affiliation relationships.
(2) Constructing a weighted heterogeneous patent innovation network. Since the constructed heterogeneous patent innovation network contains both patent and patentee nodes, it is necessary to use a weighted mechanism to distinguish the weight of different types of nodes. In this study, the weight of jumping from a patent to its reference patent is set as β, while the weight of jumping to its patentee is (1-β), and if there are more than one patents or patentees, the weight is averaged. As can be seen in Fig. 1, patent $p_{1}$ has two reference patents $p_{2}$ and $p_{3}$, so its weight to both is β/2. Additionally, patent $p_{1}$ has only one patentee $a_{1}$, so the weight is (1-β). Since the target node for the patentee can only be a patent, its weight remains at 1.
(3) Calculating three types of network node characteristic values, including the value of the patentee's credit allocation, the patentee's H index, and the time decay factor of the patent. As shown in Fig. 1, the credit values of the two patentees $a_{1}$ and $a_{2}$ in patent $p_{3}$ are first calculated, where the credit value of patentee $a_{1}$ is 0.66, and the patentee $a_{2}$ is 0.34. Secondly, the H index of the patentee is calculated, such as the H index of patentee $a_{1}$ is 5. Finally, the time decay factor of the patent is calculated based on the date of application and the date of assessment, and from which f(age) is calculated.
(4) Using a biased restart random walk model to evaluate the impact of patentees. The above three types of node features are added to the PageRank algorithm to perform the random walk process to calculate each patentee's impact score. Finally, patentees are ranked according to their scores, as shown in Fig. 1, the patentees are ranked as $a_{1}$ and $a_{2}$. As the ranking of $a_{1}$ is higher than that of $a_{2}$, it indicates that the impact of patentee $a_{1}$ is higher than that of patentee $a_{2}$.

### 3.2 Constructing a heterogeneous patent innovation network

Definition: (heterogeneous patent innovation network) Given a network G=(V,E), where V denotes the nodes of the network including patents and patentees, and E denotes the edges of the network including citation relationships between patents, affiliation relationships between patents and patentees. Given a set of patent $p=\left\{p_{1},p_{2},...,p_{m}\right\}$ and a set of patentee $a=\left\{a_{1},a_{2},...,a_{n}\right\}$, let $E_{\text{PP}}$ denotes the citation links between patents, and $E_{\text{PA}}$ denotes the affiliation links between patents and patentees. Therefore, the heterogeneous patent innovation network is represented as a graph $G=\left(a\cup p,E_{\text{PP}}\cup E_{\text{PA}}\right)$, and we hope to get a vector a from graph G that can reflect the importance of patentees.

If the heterogeneous patent innovation network contains *m* patents and *n* patentees, the graph can be represented by a binary (m+n)×(m+n) adjacency matrix A:

(1) $$A=\left(\begin{array}{cc} A_{PP} & A_{AP}\\ A_{PA} & 0 \end{array}\right)$$

Where $A_{PP}$ is the citation matrix between patents, $A_{PA}$ and $A_{AP}$ represent patent-patentee relationships where $A_{PA}=A_{AP}^{T}$, and $a_{ij}=1$ if node *i* points to node *j*. It is significant to note that there is no direct relationship between patentees in the graph.

**Fig. 2:**
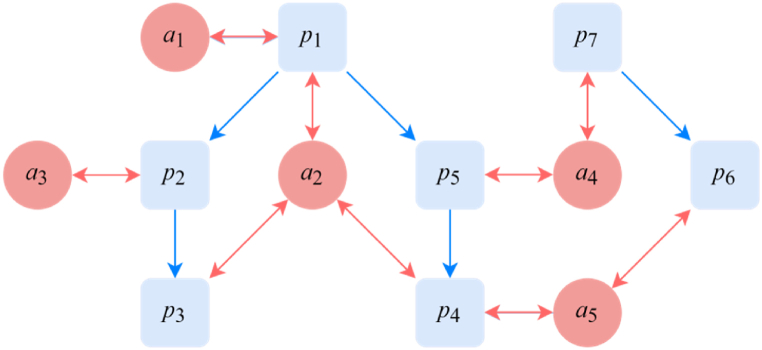
Example of the heterogeneous patent innovation network.

Fig. 2 gives a structural example of a heterogeneous patent innovation network, which is distinct from other networks such as patent-subject networks [[24], [25], [26]] and academic paper-author networks [[27], [28], [29]]. In our proposed network, there are no direct links between patentees. The is due to several reasons: (1) Relationships between patent subjects can be derived from multiple sources, such as cooperative joint patents and citation relationships between patentees. These derivative relationships often result in loss of information during graph transformation, and the number of derived relationships can increase exponentially. For example, if a patentee has applied for *m* patents, each citing an average of *n* patents and containing *k* co-patentees, then the number of citation relationships between patentees would be increase drastically to m×n×k. (2) Direct links between patentees can lead to social network dominance in the ranking system, whereby a small group of co-patentees of a patent forms and receives more traffic, resulting in a higher ranking of group members. (3) The ranking of the patentee should be determined by patents, not by the patentee's social relationships. Therefore, we exclude the direct links between co-patentees in constructing the heterogeneous patent innovation network.

Next, we analyze the constructed heterogeneous patent innovation network. To achieve this, we define the graph adjacency matrix ***A*** in Eq. (1), which is then transformed into a column random matrix ***B*** while ensuring that the sum of each column is 1. By doing so, we can consider the network as a Markov chain process and represent the impact of the patentee as a smooth distribution of the Markov process. In order to achieve the eventual stabilization of the Markov chain, we introduce a new stochastic matrix ***M*** and add a virtual link with equal but small transition probability to each pair of nodes:

(2) $$M=\alpha B+\left(1-\alpha \right)\frac{1}{n}ee^{T}$$

Where e denotes a unit vector with a n-dimension, α is the damping factor, *n* denotes the dimensions of a matrix. Therefore, the Markov process represented by stochastic matrix ***M*** is guaranteed to be strongly connected and non-cyclical, and its stable distribution is guaranteed as well. And the ranking of patentee or patent is the main eigenvector ***r*** of matrix ***M***, which can be calculated by Eq. (3).

(3) Mr=r

**Fig. 3:**
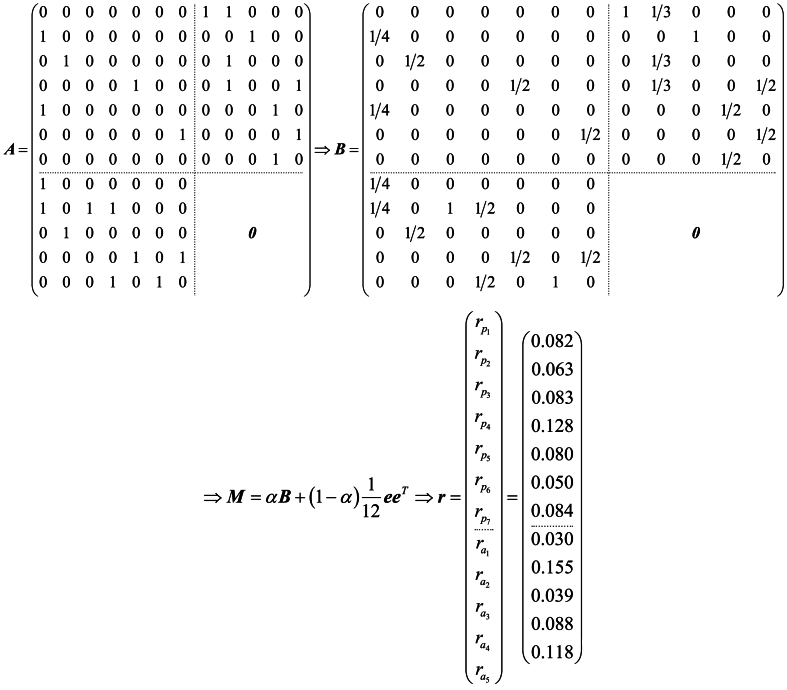
Solutions process of the heterogeneous patent innovation network (Fig. 2 as an example).

We use Fig. 2 as an example of the heterogeneous patent innovation network consisting of seven patents and five patentees, including citations between patents and affiliations between patents and patentees. To analyze this network, we transform it into a graph adjacency matrix ***A***, then transform ***A*** into a column random matrix ***B***, and add virtual contiguity to create a new random matrix ***M***. With a damping factor α=0.85, the importance of the patentee and patent is determined by the main eigenvector ***r*** of matrix ***M***. The results in Fig. 3 indicate that patentee $a_{2}$ has greater influence as it has invented three patents and one of its patent contains a citation relationship. Moreover, patentee $a_{1}$ and $a_{3}$ are contained only one patent, but since patentee $a_{3}$ has a patent $p_{2}$ is cited once, hence it is more important than patentee $a_{1}$.

$$A=\left(\begin{array}{cc} \begin{array}{ccccccc} 0 & 0 & 0 & 0 & 0 & 0 & 0\\ 1 & 0 & 0 & 0 & 0 & 0 & 0\\ 0 & 1 & 0 & 0 & 0 & 0 & 0\\ 0 & 0 & 0 & 0 & 1 & 0 & 0\\ 1 & 0 & 0 & 0 & 0 & 0 & 0\\ 0 & 0 & 0 & 0 & 0 & 0 & 1\\ 0 & 0 & 0 & 0 & 0 & 0 & 0 \end{array} & \begin{array}{ccccc} 1 & 1 & 0 & 0 & 0\\ 0 & 0 & 1 & 0 & 0\\ 0 & 1 & 0 & 0 & 0\\ 0 & 1 & 0 & 0 & 1\\ 0 & 0 & 0 & 1 & 0\\ 0 & 0 & 0 & 0 & 1\\ 0 & 0 & 0 & 1 & 0 \end{array}\\ \begin{array}{ccccccc} 1 & 0 & 0 & 0 & 0 & 0 & 0\\ 1 & 0 & 1 & 1 & 0 & 0 & 0\\ 0 & 1 & 0 & 0 & 0 & 0 & 0\\ 0 & 0 & 0 & 0 & 1 & 0 & 1\\ 0 & 0 & 0 & 1 & 0 & 1 & 0 \end{array} & 0 \end{array}\right)\Rightarrow B=\left(\begin{array}{cc} \begin{array}{ccccccc} 0 & 0 & 0 & 0 & 0 & 0 & 0\\ 1/4 & 0 & 0 & 0 & 0 & 0 & 0\\ 0 & 1/2 & 0 & 0 & 0 & 0 & 0\\ 0 & 0 & 0 & 0 & 1/2 & 0 & 0\\ 1/4 & 0 & 0 & 0 & 0 & 0 & 0\\ 0 & 0 & 0 & 0 & 0 & 0 & 1/2\\ 0 & 0 & 0 & 0 & 0 & 0 & 0 \end{array} & \begin{array}{ccccc} 1 & 1/3 & 0 & 0 & 0\\ 0 & 0 & 1 & 0 & 0\\ 0 & 1/3 & 0 & 0 & 0\\ 0 & 1/3 & 0 & 0 & 1/2\\ 0 & 0 & 0 & 1/2 & 0\\ 0 & 0 & 0 & 0 & 1/2\\ 0 & 0 & 0 & 1/2 & 0 \end{array}\\ \begin{array}{ccccccc} 1/4 & 0 & 0 & 0 & 0 & 0 & 0\\ 1/4 & 0 & 1 & 1/2 & 0 & 0 & 0\\ 0 & 1/2 & 0 & 0 & 0 & 0 & 0\\ 0 & 0 & 0 & 0 & 1/2 & 0 & 1/2\\ 0 & 0 & 0 & 1/2 & 0 & 1 & 0 \end{array} & 0 \end{array}\right)\Rightarrow M=\alpha B+\left(1-\alpha \right)\frac{1}{12}{ee}^{T}\Rightarrow r=\left(\begin{array}{c} r_{p_{1}}\\ r_{p_{2}}\\ r_{p_{3}}\\ r_{p_{4}}\\ r_{p_{5}}\\ r_{p_{6}}\\ r_{p_{7}}\\ r_{a_{1}}\\ r_{a_{2}}\\ r_{a_{3}}\\ r_{a_{4}}\\ r_{a_{5}} \end{array}\right)=\left(\begin{array}{c} 0.082\\ 0.063\\ 0.083\\ 0.128\\ 0.080\\ 0.050\\ 0.084\\ 0.030\\ 0.155\\ 0.039\\ 0.088\\ 0.118 \end{array}\right)$$

### 3.3 Construct a weighted heterogeneous patent innovation network

**Fig. 4:**
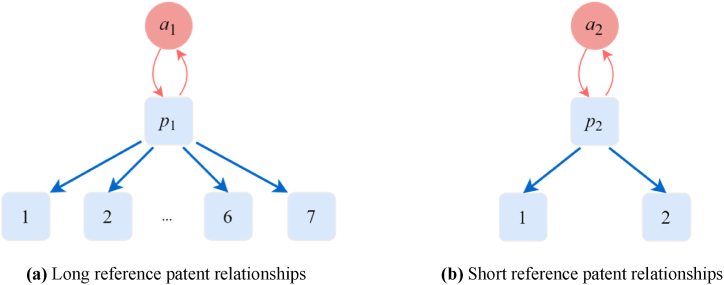
Problem with long reference in the heterogeneous patent innovation network.

Although constructing a heterogeneous patent innovation network and assessing the impact of patentees using the common PageRank algorithm is feasible, it lacks the ability to distinguish between the types of nodes in the network, which is an unreasonable assumption. A problem with long reference citations for patents in the constructed network was discovered, as shown in Fig. 4. In this example, Fig. 4(a) shows that patent $p_{1}$ is patented by $a_{1}$ and contains seven references; while Fig. 4(b) shows that patent $p_{2}$ is patented by $a_{2}$ and contains only two references. Consequently, $a_{1}$ receives a weight of 1/8 from $p_{1}$, and $a_{2}$ receives a weight of 1/3 from $p_{2}$. If $p_{1}$ and $p_{2}$ are of equal importance, then $a_{2}$ would be deemed more important than $a_{1}$. However, for patents that require more patent references for innovation, the corresponding patentees would have a very small weight. To avoid this long reference problem, a weighted mechanism is used to distinguish the weight of the patent and patentee. Specifically, we set the jump rate from the patent to its reference patent as β, and the jump rate to the relevant patentee as (1−β). If there are more than one reference patent or patentee, the jump rate is divided equally. Since the connection node of the patentee can only be a patent, the jump rate of the patentee to patent is not considered. Consequently, we obtain a new column stochastic matrix ***B***, in which the sum of the weights of each column is still 1.

(4) $$B=\left(\begin{array}{cc} \beta \cdot A_{PP}^{\prime } & A_{AP}^{\prime }\\ \left(1-\beta \right)\cdot A_{PA}^{\prime } & 0 \end{array}\right)$$

Taking Eq. (4) into Eq. (2) and Eq. (3), and calculating the importance score for patentees and patents.

### 3.4 Calculating node characteristics in the heterogeneous patent innovation network

The heterogeneous patent innovation network comprises nodes of different types, each with its own unique properties. To enhance the accuracy of patentee impact assessment, we also take into account the specific information of nodes in the network, such as the value of patentee's credit allocation, the patentee's H index, and the patent's time decay factor. Credit allocation value of the patentee reflects its reputation and significance in the academic or industry domain [30,31]. The H index is a scientometric index used to assess the personal contribution and impact of scientists [32,33]. The time decay factor of a patent considers the age factor of the patent. Over time, the impact of a patent typically diminishes gradually [34,35]. Therefore, these three types of characteristics play a significant role in assessing the impact of patentees. Credit allocation value and H index reflect the reputation and contributions of patentees in the academic or industry domains, while the time decay factor considers the timeliness of patents. By comprehensively considering these characteristics, a more accurate assessment of the impact of patentees can be achieved, thereby providing strong support for relevant decision-making and research.

(1) Credit allocation value of the patentee. Patentees can collaborate to combine ideas and technologies from different fields, leading to the creation of more innovative and high-quality patents. In the process of inventing a patent, multiple patentees may work together, each with their own contribution. It is necessary to allocate the value of the contribution of the different patentees to the same patent.

Previous studies have commonly used simple methods to allocate credit value to patentees. For patents with a single patentee, a credit value of 1 is assigned. However, for patents with multiple patentees, the fractional counting method is used to distribute the credit value equally among *N* patentees, where each patentee receives a credit value of 1/*N* [30]. While this method is criticized for its unfair distribution. To solve this problem, scholars have explored various methods such as geometric, harmonic, arithmetic, and network-based methods, taking into account the order of patentees. For instance, the harmonic method allocates a score to all patentees of a patent, where the credit value of patentee *i* is proportional to the inverse of their ranking in the list. Thus, the higher the ranking of the patentee, the higher their credit value. In a patent with three patentees in the order of A, B, and C, the credit value of patentee A is 1/(1 + 1/2 + 1/3) = 6/11, and the credit values of patentees B and C are 3/11 and 2/11, respectively. This method considers the actual contribution of the patentee based on their order of authorship and has some rationality.

**Fig. 5:**
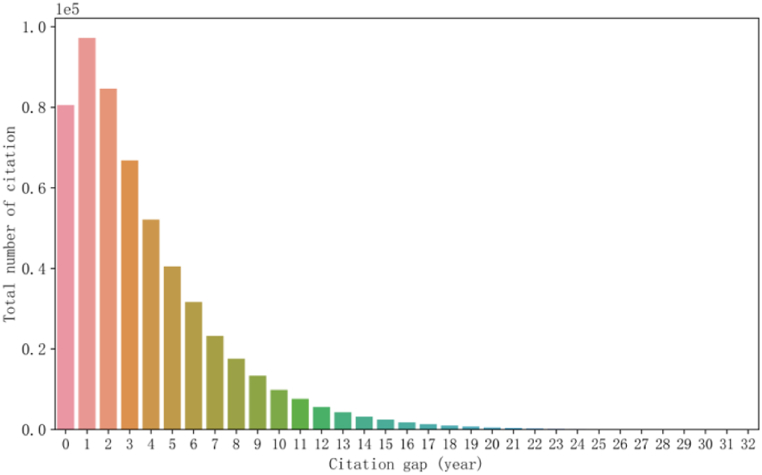
Patent citation gap and the total number.

(2) H index value of the patentee. The H index is an evaluation method based on citation and is widely used in academia to assess an author's research impact [3]. In this study, we propose to incorporate the H index into the PageRank algorithm to increase the probability of random access for patentees and enhance their influence. The H index of a patentee refers to the number of their patents that have been cited at least H times, with the assumption that patentees with higher H index have greater influence. Hence, we employ the H index calculation method to analyze the H index of patentees, which constitutes a crucial factor in quantifying the impact of patentees.
(3) Time decay factor of the patent. This study revealed that newer patents were more likely to be cited and that recent technological innovations received greater attention. Fig. 5 shows the total number of citations for patent reference intervals in the green innovation patent dataset created in this paper. The total number of citations per patent increased over time, with the number of annual citations for a patent increases over the 0–1 year following the patent application, followed by a yearly decrease. Thus, to accurately measure the impact of a patent, more weight should be given to recent patents, with consideration of the timing of the patent application. We introduce the time decay factor of a patent in the calculation of its impact score, taking into account the evolution of its value over time. The effect of the citation timeliness of a patent on its value can be transmitted through its reference relationships.

To calculate the time decay factor of a patent, we utilize the application year and evaluation year of the patent. In this study, we use the time decay function $f\left(age_{i}\right)$ as shown in Eq. (5), where $T_{a}$ denotes the year of assessment, $T_{p}$ denotes the year of patent application, and ρ is the parameter that controls the extent of time decay. Eq. (5) is a monotonically decreasing function, indicating that the time decay factor of a patent decreases with the length of time since its publication. This approach allows for a more accurate assessment of the impact of a patent by considering its timeliness in relation to the evaluation period.

(5) $$f\left(age_{i}\right)=e^{-\rho *\left(T_{a}-T_{p}\right)}$$

### 3.5 Biased restart random walk model to evaluate the impact of patentees

To incorporate fine-grained information of nodes in the network, we introduce the three types of node characteristics obtained above into PageRank algorithm, and implement the random walk process in the weighted heterogeneous patent innovation network. The improved algorithm (name *CWAPN*) not only distinguishes different types of nodes using a weighted mechanism and jump rate, but also weights the intrinsic properties of nodes. This approach more rationally simulates the assessment process of nodes in heterogeneous patent innovation networks. The *CWAPN* algorithm calculates the random walking score of patentees based on the following two assumptions.

(1) The impact score of a patentee is affected by the score of all patents he has applied for. This indicates that the impact of a patentee in the field is manifested through the quality of patents invented.
(2) The impact score of a patent is not only affected by the score of its belonging patentee, but also by the reference patents that cited it. For instance, patentees with a higher reputation are more inclined to invent high-quality patents. Moreover, if a patent is cited by another high-quality patent, its impact score should also be higher, indicating that the patent has significant technological innovation.

Therefore, the impact of patentees and patents in the constructed network mutually reinforces each other. Supposed the total number of patentees is $N_{a}$ and the total number of patents is $N_{p}$, initial scores of patentees and patents are both set to $1/\left(N_{a}+N_{p}\right)$. The random walk model for calculating the impact of patentees in the heterogeneous patent innovation network is shown below:

(1) Update the PageRank score of the patentee by Eq. (6).

(6) $$\mathrm{PR}\left(a_{i}\right)=\left(1-\alpha \right)*\frac{hindex\left(a_{i}\right)}{\sum_{j=1}^{N_{a}}hindex\left(a_{j}\right)}+\alpha *\left(1-\beta \right)\sum_{j=1}^{m}\left(\mathrm{PR}\left(p_{j}\right)*credit\left(a_{i}\right)\right)$$

where $\mathrm{PR}\left(a_{i}\right)$ is the PageRank score obtained by $a_{i}$ in the network, $hindex\left(a_{i}\right)$ is the H index of patentee $a_{i}$, α is the damping factor, (1−β) denotes the weight of the jump rate from patent to patentee, $\mathrm{PR}\left(p_{j}\right)$ is the PageRank score obtained by the patent $p_{j}$ in the network, $credit\left(a_{i}\right)$ is the credit allocation value of patentee $a_{i}$ in patent $p_{j}$, and m denotes the total number of patents owned by patentee $a_{i}$. Eq. (6) is composed of two parts, the first part is the restart probability value of patentee $a_{i}$, which reflects that the patentee's impact is greater with a higher H index; the second part is the weighted probability of jumping from the patentee's neighbor (patent) to the patentee, which takes into account the patentee's credit allocation in each patent. The higher the ranking of the patentee, the higher their credit rating.

(2) Update the PageRank score of the patent by Eq. (7).

(7) $$\mathrm{PR}\left(p_{j}\right)=\left(1-\alpha \right)*\frac{f\left(age_{j}\right)}{\sum_{s=1}^{N_{p}}f\left(age_{s}\right)}+\alpha *\left[\sum_{i=1}^{n}\frac{\mathrm{PR}\left(a_{i}\right)}{C\left(a_{i}\right)}+\beta *\sum_{i=1}^{w}\frac{\mathrm{PR}\left(p_{i}\right)*f\left(age_{i}\right)}{L\left(p_{i}\right)}\right]$$

Where $f\left(age_{j}\right)$ is the time decay factor of patent $p_{j}$, $N_{p}$ is the total number of patents in the network, $C\left(a_{i}\right)$ is the total number of patents owned by patentee $a_{i}$, *n* denotes the number of patentees included in $p_{j}$, *w* is the number of citations in $p_{j}$, and $L\left(p_{i}\right)$ is the total number of reference patents for $p_{j}$. Eq. (7) is composed of three parts, the first part uses the decay factor of patent's application time to weight the restart probability of $p_{j}$. If the patent is relatively recent, the value of $f\left(age_{j}\right)$ is higher, indicating that the patentee is more likely to cite a recently filed patent in their patent citations; the second part indicates the probability of the patentee $a_{i}$ reaching the patent $p_{j}$, and the impact score of the patent is influenced by the impact score of the associated patentee; the third part represents the probability of jumping from neighboring patents to the target patent, and its impact score is influenced by the impact scores of its reference patent.

(3)Repeat the above two steps until the PageRank scores of all patentees and the PageRank values of all patents converge. The convergence condition is that the sum of the difference values obtained from the two iterations of all patents and patentees is less than a fixed threshold value. Therefore, the pseudo-code for the implementation of the *CWAPN* algorithm proposed is shown in **Algorithm 1**, where the input $G_{AP}$ denotes the constructed heterogeneous patent innovation network, init_assignee(A) is the initial PageRank value of the patentee, init_patent(P) is the initial PageRank value of the patent, credit(P) is the credit allocation value of patentee of the patent in the network, fages(P) is the time decay factor of the patent, hindex(A) is the innovation degree of the patentee, and threshold is the error threshold. The output $PR_{k}\left(A\right)$ is the final PageRank value of the patentee, which indicates the impact score of the patentee.

**Figure:**
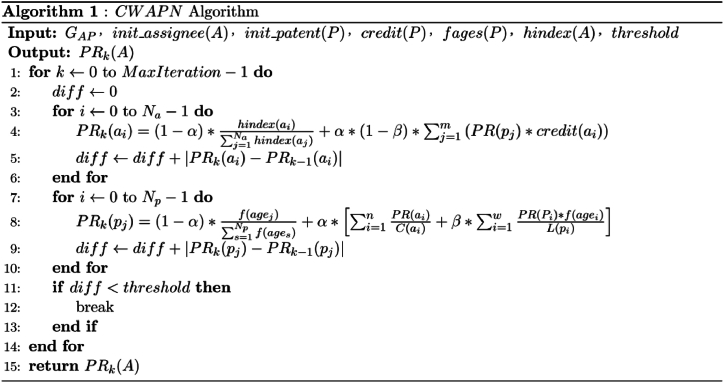

The impact score of each patentee is calculated using **Algorithm 1** and then sorted in descending order based on the score values to obtain the actual impact ranking of each patentee.

## 4 Data and methods

This section presents the description of data and methods used in our study. We constructed a dataset of green innovation patents in China and provided a comprehensive explanation of the baseline algorithms relevant to patentee impact. To assess the impact of patentee, we employed *NDCG* and *RI* as evaluation indicators, providing a research foundation for investigating the performance of the *CWAPN* algorithm on a real-world dataset.

### 4.1 Green innovation patent dataset

The granted patents used in this study were obtained from the website of the China National Intellectual Property Administration (CNIPA).^1^ We specifically selected granted patents instead of other types of patents, as previous researches have shown that granted patents are a better indicator of the quality and innovative capacity of patents [36,37]. Then, the “green list of international patent classification^2^” published by the World Intellectual Property Organization (WIPO) was used to identify green granted patents in China. Furthermore, patent citation information was gathered from the Google Patents website^3^ using the patent granted number. Therefore, the green innovation patent dataset for this study was obtained, with a time dimension of 1985–2020 and a total number of 539,297 patents.

**Fig. 6:**
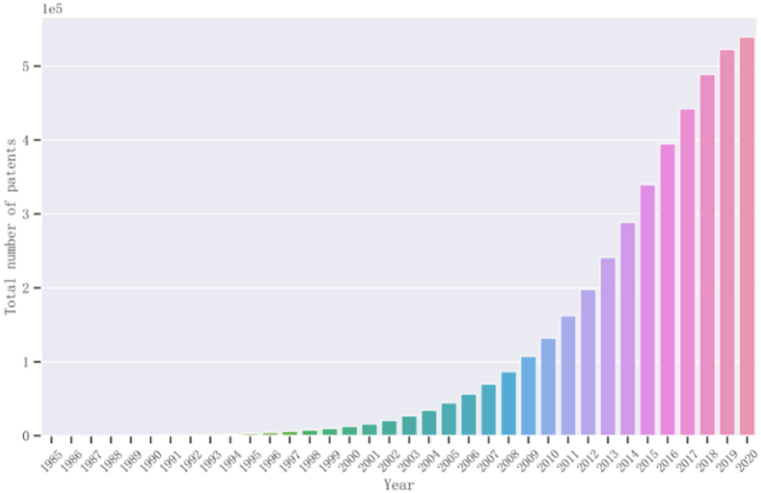
Cumulative annual number of green granted patents in China. (For interpretation of the references to colour in this figure legend, the reader is referred to the Web version of this article.)

Fig. 6 illustrates the cumulative annual number of green granted patents in China. It indicates that the quantity of green patents was relatively small before the year 2000, with a sharp exponential increase observed from 2001 to 2018, which demonstrates the growing interest in green technological innovation. Therefore, by examining Chinese green patents, important and influential patentee information can be extracted. This information aids in a comprehensive understanding of the market competition in the green innovation field, and provides objective data support for green innovation management, policy formulation, and strategic decision-making. Ultimately, this contribution can help achieve green development and double carbon targets in China.

To ensure high-quality research data, patent datasets must undergo cleaning and preprocessing to remove noise data, such as identifying and consoling unreasonable patent citation relationships and resolving previous name inconsistencies in the patentee list. After these steps, obtained the information contained in the constructed heterogeneous patent innovation network, which is presented in Table 2.

**Table 2:** Summary of data characteristics of the heterogeneous patent innovation network.

Data field	Data value
Patent nodes	539,297
Patentee nodes	119,539
Patent-patent edges	640,113
Patent-patentee edges	631,168

### 4.2 Baseline algorithms

There is no uniform measure for assessing the impact of technological innovation, we rely on the ranking of citation count (excluding self-citations) against patentees over the past three years as a base fact to verify the algorithm's performance. Consistent with prior work by Chen et al. [38], we employ the PageRank algorithm to assess node importance and set the damping factor α=0.5 for citation networks. We utilize a random walk model and execute 1000 iterations to ensure convergence, setting the stopping threshold at 1e-8. To validate the proposed algorithm, we compare it against several baseline algorithms.

(1) *G*: This algorithm represents the *G* index of a patentee, which is a derivative of the *H* index widely used to assess the quantity and quality academic output among researchers. The *G* index has been adopted by many researchers to evaluate the impact of patent fields [13].
(2) *PN_SPR*: This algorithm represents the constructed of a patent citation network based on the relationships between patents, followed by the application of the PageRank algorithm to calculate importance scores for each patent. The scores are then summed up to obtain the influence score of the corresponding patentees.
(3) *TAPRW*: This algorithm is employed to assess the impact of scholars in an academic network. Firstly, it calculates the PageRank score of the paper's time perception in the citation network, and utilizes it as the initial value for random walk in the heterogeneous author-paper network. The constructed heterogeneous patent innovation network is similar to this network, this algorithm is also used as a baseline algorithm.
(4) *FutRank*: FutureRank is a novel algorithm that leverages both the connection structure between nodes and the time series information of nodes in a network to predict their influence [39]. This algorithm first applies the PageRank algorithm to the patent citation network, followed by the HITS algorithm on the patent-patentee network, and combines their results. Notably, it introduces a function that incorporates the patent application time, as it is observed that patentees are more likely to cite recently filed patents. This study utilizes the FutureRank algorithm to analyze the influence of patentees, and it is presented as a comparative algorithm in this study.
(5) *WAPN*: This algorithm represents the weighted heterogeneous patent innovation network constructed in this study. The weighted mechanism is employed to distinguish different types of nodes, and the problem of long reference citation in patents is solved to obtain a relatively objective assessment of patentee impact.

### 4.3 Evaluation indicators

Assessing the impact of patentees can help in discovering key technologies and innovative subjects for core patents in the technology field. It can also reveal linkages between patentees and provide decision-making support for promoting R&D cooperation, technology research, and development strategies. Therefore, it is crucial to identify high-ranked patentees in the impact assessment algorithm of technological innovation.

However, most previous literature ranking studies face a common challenge of effectively evaluating the ranking algorithm's results due to the lack of ground-truth data. Citation count is widely accepted as a measure to determine the impact of researchers or patentees. A high impact score indicates a certain level of prestige in the relevant technology field. Specifically, the number of citations (excluding self-citations) received by a patentee's invention in a future period is used to reflect the patentee's impact. In this study, we divided the dataset into two parts: the ranking part and the evaluation part. We use the Chinese green patent dataset spanning from 1985 to 2017 to implement these ranking algorithms, and the sum of all citations of the patentee from 2018 to 2020 was used as the patentee's impact, which served as the ground-truth ranks for the *FutCit* algorithm.

By comparing the *CWAPN* algorithm with baseline algorithms, *NDCG* and *RI* indicators are used to evaluate the precision and ranking performance of the algorithm's performance. The reasons why *NDCG* is suitable for evaluating algorithms of assessing the impact of network nodes are as follows: (1) It considers the quality of rankings and measures the similarity between algorithmic rankings and true influence rankings. (2) It emphasizes high-influence nodes by assigning greater weight to nodes ranked higher, effectively capturing nodes with significant influence. (3) *NDCG* exhibits good interpretability. With values ranging from 0 to 1, a higher value indicates that the algorithmic rankings are closer to the true influence rankings, making it easy to understand and interpret the evaluation results. In summary, *NDCG* has the advantages of considering ranking quality, emphasizing high-influence nodes, and being interpretable, making it highly suitable for evaluating algorithms for assessing the influence of network nodes. Additionally, *RI* is applicable for assessing the precision of ranking algorithms in recommendation systems. This indicator is used to measure the confidence or strength of the recommendations provided by the system. In recommendation systems, recommendations are often ranked based on their intensity scores, which reflect the system's confidence in the user's potential interest in the recommended items. Higher intensity scores are assigned to items the system is more certain about, leading to their higher positions in the ranked list, as the system believes users are more likely to be interested in them. Consequently, *RI* not only considers ranking precision but also emphasizes recommendations with higher confidence by giving them greater weight. Therefore, using this indicator to assess the precision of ranking algorithms is also reasonable. The specific evaluation steps are as follows: (1) The ranking list of patentees obtained by the *FutCit* algorithm is treated as the ground-truth. (2) Different node impact assessment algorithms are applied to calculate the importance scores of patentees. (3) Nodes are sorted based on their importance scores, resulting in the algorithm's node ranking list. (4) The algorithm's node ranking list is compared with the ground-truth to compute the *NDCG* and *RI* scores, thus facilitating a comprehensive performance analysis of different influence evaluation algorithms.

(1) Normalized discounted cumulative gain (*NDCG*) is commonly used as a measure of ranking quality. It considers not only the relevance of ranking lists but also the location of ranking results. The *NDCG* incorporates two crucial ideas into its assessment: ① It rates the relevance of a patentee's list by ranking it, where a higher ranking indicates greater importance of the patentee. ② It assigns a higher score to a highly correlated result occurring in a higher ranking position, just as show in Eq. (8).

(8) $$NDCG@k=\frac{DCG@k}{IDCG@k}=\frac{\sum_{i=1}^{k}\frac{2^{rel_{i}}-1}{\log_{2}\left(i+1\right)}}{\sum_{i=1}^{\left|REL\right|}\frac{2^{rel_{i}}-1}{\log_{2}\left(i+1\right)}}$$

where $rel_{i}$ denotes the relevance of the *i-*th result; REL denotes the ground-truth list length in top-*k* after ranking the relevance in descending order; DCG@k denotes the top-*k* nodes in the ranking, where the relevance of $rel_{i}$ is considered node by node and multiplied by a discounted value $1/\log_{2}\left(i+1\right)$; IDCG@k is the maximum DCG value in the ideal case, which normalizes the value of DCG@k. In this study, the value of $rel_{i}$ is set to 1 if the node is in the top-*k* of the ground-truth list. Otherwise, it is set to 0. Therefore, The NDCG indicator not only considers the relevance of results, but also incorporates the position of the results within the ranking list, which makes it more accurate in evaluating the quality of rankings.

The result of *NDCG* ranges from 0 to 1. When *NDCG* = 0, it means that the algorithm's ranking result is completely inconsistent with the ground-truth ranking, indicating poor algorithm performance. On the other hand, when *NDCG* = 1, it indicates that the algorithm's ranking result perfectly matches the ground-truth ranking, demonstrating optimal algorithm performance. By observing the *NDCG* scores, we can intuitively understand the performance of the ranking algorithm. Higher *NDCG* scores imply that the algorithm can accurately predict influential patentees. Conversely, lower *NDCG* scores necessitate further optimization of the algorithm to enhance the precision and quality of the ranking.

(2) Recommendation intensity (*RI*). The *RI* indicator relies on two fundamental intuitions: given two sets of ranking results R1 and R2 on their top-*k* list, the superiority of R1 over R2 can be inferred if ① R1 contains a large number of patentees that match the ground-truth ranking, and ② the patentees that match the ground-truth ranking in R1 must occupy higher positions in the top-*k* list as compared to R2 [40]. If R is the list of top-*k* patentees returned by a ranking algorithm, and *L* is the list of ground-truth entities. For each patentee *P*_*i*_ in R, with its corresponding ranked order $o_{r}$, the *RI* of *P*_*i*_ at *k* can be formally defined as Eq. (9).

(9) $$RI\left(P_{i}\right)@k=\left\{ \begin{array}{cc} 1+\left(k-o_{r}\right)/k & P_{i}\in L\\ 0 & P_{i}\notin L \end{array}\right.$$

This implies that if the patentee *P*_*i*_ is present in the top-*k* ground-truth list R and is ranked higher (i.e., has a smaller $o_{r}$ value), its corresponding *RI* value will be higher. By aggregating the *RI* value of each patentee in the list R, the overall *RI* of the list at *k* can be defined as Eq. (10).

(10) $$RI\left(R\right)@k=\sum_{P_{i}\in R}RI\left(P_{i}\right)@k$$

Recommendation intensity shares some similarity with precision at *k* in the context of information retrieval. Specifically, if we consider the top-*k* recommendations in list *R* to be unordered and divide the value of RI, denoted by $RI\left(P_{i}\right)@k$ at *k*, then it reduces to precision at *k*.

The result of *RI* are associated with the chosen value of *k*. By fixing the value of *k*, a comparison of *RI* scores obtained from ranking algorithms can effectively assess their performance. A higher *RI* score indicates a stronger recommendation strength of the algorithm, signifying higher confidence in the recommended results. Consequently, it facilitates finding influential patentees.

## 5 Results and analysis

Table 3 displays the top 20 patentees of each ranking algorithm in the Chinese green patent dataset, where “Patentee” denotes the name of the patentee in English, and “Rank” signifies the attained ground-truth ranking. Meanwhile, we computed the Pearson correlation coefficients for the rankings of the top 20 patentees in the ground truth list compared to each ranking algorithm.

**Table 3:** Ranking of top 20 patentees in the Chinese green patent dataset.

Patentee	Rank	*G*	*PN_SPR*	*TAPRW*	*FutRank*	*WAPN*	*CWAPN*
State Grid Corporation of China	1	9	4	1	1	1	1
Tsinghua University	2	11	2	4	4	4	4
Southeast University	3	6	6	6	6	6	6
Zhejiang University	4	15	5	5	5	5	5
North China Electric Power University	5	54	15	10	12	14	9
China Electric Power Research Institute	6	11	17	16	18	19	10
South China University of Technology	7	29	10	7	9	9	7
Shanghai Jiao Tong University	8	25	7	8	10	7	8
Chongqing University	9	25	19	19	23	18	18
China Petroleum and Chemical Corporation	10	2	1	2	2	2	2
Xi'an Jiaotong University	11	54	20	20	20	21	19
Tongji University	12	29	21	22	22	20	21
Harbin Institute of Technology	13	69	13	13	13	11	14
Shandong University	14	54	26	25	27	26	23
Huazhong University of Science and Technology	15	95	31	29	31	29	29
Beihang University	16	25	35	37	43	36	36
Tianjin University	17	29	18	18	17	17	17
Hohai University	18	54	23	21	21	22	16
Wuhan University	19	69	39	39	44	43	40
Guangdong OPPO Mobile Telecommunications Corp., Ltd.	20	4	134	103	94	122	76
Correlation coefficient	–	0.46	0.63	0.70	0.73	0.65	**0.76**

**Fig. 7:**
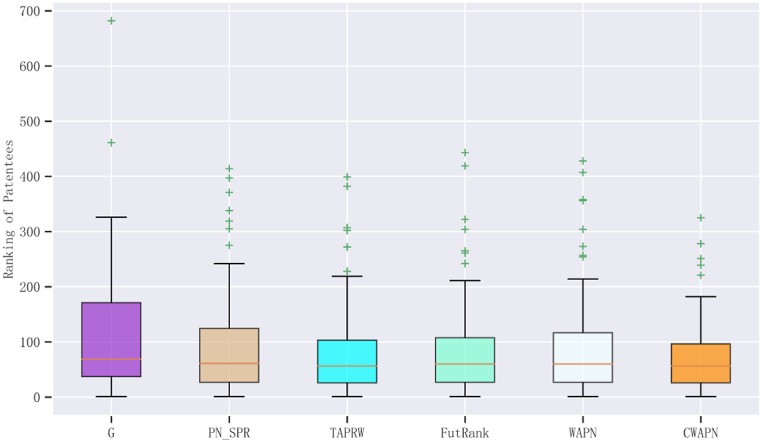
Box plots of the ranking positions for top 100 patentees.

According to Table 3, among the top 20 ranked patentees in the Chinese green patent dataset, 16 are universities, 3 are enterprises and 1 is a research institute. This illustrates that the primary contributors to green technological innovation in China are reputable universities, authoritative enterprises and research institutions. Additionally, a comparison between the ground-truth rankings and the rankings obtained by various algorithms reveals that the rankings by *CWAPN* algorithm closely aligns with the ground-truth rankings. This suggests that the proposed algorithm can more accurately identify influential patentees. By examining the Pearson correlation coefficients of the rankings for the top 20 patentees derived from the ground truth list and various algorithms, it becomes evident that the ranking produced by the *CWAPN* algorithm closely aligns with the ground truth rankings, which has a score of 0.76. This finding provides further substantiation for the exceptional performance of the *CWAPN* algorithm. Moreover, in order to better demonstrate the superiority of the *CWAPN* algorithm, the top 100 ranking results are presented in box plots, as shown in Fig. 7.

From the above box plots, we can see the lowest, highest, and mid-range rankings of the top of 100 patentees, with "+" representing outliers in the ranking positions. It can be seen that the ranking results of the *G*, *PN_SPR* and *WAPN* algorithms are relatively scattered, and the rankings of *TAPRW*, *FutRank* and *CWAPN* algorithms are relatively concentrated. As the ground truth ranking results range from 1 to 100, the closer the results of different ranking algorithms are, the smaller the variability of the data, indicating a lower level of data dispersion. This can be interpreted as the data points being relatively close in the statistical distribution and concentrated with a narrow range. When the box plot displays more concentrated results, it generally indicates: (1) relative stability in data distribution without many outliers, (2) higher consistency among different samples or observations with small differences, and (3) higher reliability, as the data concentration implies more credible results and greater accuracy in the algorithm's outcomes. Therefore, our analysis using the box plot reveals that the *CWAPN* algorithm produces ranking results that are highly concentrated, with the majority falling within the top 100. This further confirms the algorithm's ranking closely aligns with the ground truth ranking. And from the upper quartile, maximum values of the box plot, the proposed *CWAPN* algorithm also has a smaller ranking than *TAPRW* and *FutRank* algorithm, which shows that *CWAPN* algorithm is more effective than other ranking algorithms.

### 5.1 NDCG

Table 4 represents the *NDCG* results for patentees ranked in the top *k* positions, with *k* equals to 10, 20, 50, 100, 200, 300, and the average *DNCG* within the top 300 were obtained using assessment algorithms in the heterogeneous patent innovation network. The table clearly illustrates that our proposed *CWAPN* algorithm significantly outperforms all baseline algorithm at different top *k* rankings. Moreover, the *CWAPN* algorithm exhibits the highest average *NDCG* of the top 300 rankings. It is 25.42 % higher than the *G* index, 6.34 % higher than the *PN_SPR* algorithm and 2.90 % higher than the *FutRank* algorithm. These results further demonstrate the *CWAPN* algorithm's ability to accurately identify influential patentees.

**Table 4:** Top *k* of *NDCG* in the heterogeneous patent innovation network (%).

Algorithm	Top 10	Top 20	Top 50	Top 100	Top 200	Top 300	Average 300
*G*	39.38	48.78	59.68	69.38	65.27	64.10	62.75
*PN_SPR*	77.02	75.64	78.95	76.28	73.97	72.76	74.01
*TAPRW*	85.53	75.64	80.45	79.67	76.70	77.09	77.51
*FutRank*	77.02	71.36	78.88	78.35	75.75	76.82	76.48
*WAPN*	77.02	76.32	77.33	77.02	74.19	73.96	75.37
*CWAPN*	**93.37**	**78.98**	**82.06**	**80.94**	**78.82**	**77.64**	**78.70**

### 5.2 RI

The *RI* results of top *k* in the heterogeneous patent innovation network for patentees are shown in Table 5. The results indicate that the performance of the *CWAPN* algorithm still outperforms other baseline algorithms. Moreover, this algorithm exhibits the highest average *RI* of the top 300 rankings. It is 26.33 % higher than the *G* index, 6.18 % higher than the *PN_SPR* algorithm and 2.27 % higher than the *FutRank* algorithm. This finding suggests that the rankings generated by the CWAPN algorithm are more accurate reflections of the ground-truth rankings of patentee influence.

**Table 5:** Top *k* of *RI* in the heterogeneous patent innovation network.

Algorithm	Top 10	Top 20	Top 50	Top 100	Top 200	Top 300	Average 300
*G*	4.30	11.10	40.28	93.88	181.06	276.06	137.14
*PN_SPR*	10.50	19.15	56.18	110.70	218.01	322.25	163.16
*TAPRW*	11.70	19.55	57.38	115.66	227.26	343.39	171.47
*FutRank*	10.30	18.15	55.80	112.90	224.23	341.02	169.40
*WAPN*	10.60	19.35	55.16	112.08	219.10	328.73	165.93
*CWAPN*	**12.80**	**21.20**	**58.78**	**117.71**	**232.27**	**344.14**	**173.25**

In summary, the evaluation indicators of both *NDCG* and *RI* demonstrate that the performance of the *CWAPN* algorithm outperforms that of other baseline algorithms. This suggests that employing the *CWAPN* algorithm proposed in this paper not only produces more accurate assessments of patentee influence, but also effectively resolves recommendation issues in patent innovation networks. The algorithm facilitates the analysis of patentee's position in green innovation in China and provides valuable data support for enterprises and institutions to formulate technology research and development strategies.

To validate the algorithm's superiority, we conducted a robustness analysis of patents in the field of nuclear engineering technology, and the results are presented in Table 6. The patent dataset utilized in this experiment contains 7299 patents, with 410 relevant patentees used for prediction. The top 10 % of the data is the most valuable, hence we performed an experimental analysis on the top 10, 20, 30, and 40 ranked patentees. The results indicate that the outcomes achieved by our proposed *CWAPN* algorithm were consistently among the best. Therefore, we can assert with confidence that our research conclusions will remain robust even if we were to adopt patent datasets from other fields.

**Table 6:** Robustness analysis of patents in the field of nuclear engineering technology.

Algorithm	Top 10		Top 20		Top 30		Top 40		Average 40	
	NDCG (%)	RI	NDCG (%)	RI	NDCG (%)	RI	NDCG (%)	RI	NDCG (%)	RI
*G*	84.86	**12.2**	77.10	23.55	80.61	35.53	73.54	43.7	10.2	3.20
*PN_SPR*	71.16	9.8	86.09	24.95	81.55	34.93	78.08	44.65	10.24	3.12
*TAPRW*	78.50	10.9	86.09	25.10	84.00	36.57	79.89	46.15	10.80	3.27
*FutRank*	78.50	10.9	86.09	24.95	83.52	35.83	79.89	46.4	10.55	3.28
*WAPN*	78.50	10.8	86.09	24.95	84.00	36.07	79.95	46.15	10.49	3.23
*CWAPN*	**85.12**	**12.2**	**89.29**	**26.15**	**86.20**	**37.33**	79.85	45.95	**10.83**	**3.30**

## 6 Conclusion

This study aims to construct a heterogeneous patent innovation network by combining the heterogeneity of network structure with information diversification. The main contribution of this work is the development of a weighted mechanism that distinguishes the weight of different types of nodes in the network and the excavation of three types of fine-grained information for network nodes, this method can effectively suppress the bias problem in the citation-based impact assessment algorithms, making the ranking results more reasonable. The study employs Chinese green patents from 1985 to 2020 as the research target, and the experimental results show that the proposed *CWAPN* algorithm generates a ranking of patentees that is much closer to the actual ranking, demonstrating the algorithm's validity. Hence, the implementation of the above assessment algorithms is advantageous in identifying the crucial technologies and primary patentees who have made outstanding contributions to sustainable development in China. This holds significant practical implications and applied value towards directing and evaluating the green innovation competence of businesses or organizations.

Some limitations exist in this study: (1) Only patent and patentee information is considered in the construction of the heterogeneous patent innovation network, while the impact of inventors and technology categories on the importance of patentees is not taken into account. In the future, additional information can be added to the heterogeneous information network to construct a more comprehensive network [41]. The reputation, expertise, and experience of patent inventors directly influence the quality and innovative capability of patents, and also indirectly impact the level of influence of patentees. Inventors with high reputation and respect are often capable of creating high-quality patents, which in turn, contribute to increased citation frequency and enhance the influence of patentees. Patents in different technology categories may vary in their degree of innovation; in highly innovative technology fields, patentees are more likely to wield greater influence as these patents typically represent cutting-edge technologies and significant scientific advancements. Therefore, these patent-related factors play crucial roles in assessing the impact of patentees. However, the incorporation of such information would render the heterogeneous innovation network and impact assessment model more complex, necessitating a higher level of interpretability for the obtained results. (2) Node attribute information could influence the impact of nodes in a network. This paper only explores three significant attributes that affect patents and patentees. However, these nodes also encompass other attributes, such as the type of patentee (enterprises, universities, individuals, etc.), the average time of patent citation. These attribute values may have an impact on the results of assessing the impact of patentees. Therefore, in future research, we need to investigate more crucial node attribute information and analyze the implications of evaluating the impact of patentees based on these attributes through experimentation. (3) The most direct result of assessing the impact of patentees is to identify those with authoritative patentees. However, defining the impact of patentees is a complex task, and there is currently no unified concept. Therefore, there is a need for a standardized approach to measure the authority of patentees. In future research, it is crucial to comprehensively define authoritative patentees based on various factors, which will not only contribute to the study of influence but also benefit the entire academic community.

Defining the impact of patentee requires considering multiple important features and indicators. The impact of patentees can be assessed from the following perspectives: (1) Patent quantity. The more patents a patentee possesses, the greater their influence in the technological field. (2) Citation count. The number of times a patent is cited by other patents reflects its importance and impact. A higher citation count indicates greater influence and innovative of the patentee. (3) Citation quality. A higher number of citations from other high-impact patents signifies the patent's greater significance in the technological field. (4) Patent collaboration. Whether a patentee collaborates with other authoritative patentees also reflects their authority in the field. (5) Technological field. The technological field in which the patentee is involved can also affect their influence. In some fields, patentees may receive more attention and recognition. By considering the above features and indicators, the impact of patentees can be defined using both quantitative and qualitative methods. For instance, combining patent citation data, patent collaboration network analysis, and patent quality assessment can effectively measure the impact of patentees, making it clearer and more consistent. This enhances the persuasiveness of the research findings in this paper.

## Data availability statement

The authors will supply the relevant data in response to reasonable requests.

## CRediT authorship contribution statement

**Xipeng Liu:** Writing – original draft, Visualization, Validation, Supervision, Software, Resources, Project administration, Methodology, Formal analysis, Data curation, Conceptualization. **Xinmiao Li:** Writing – review & editing, Validation, Funding acquisition.

## Declaration of competing interest

The authors declare that they have no known competing financial interests or personal relationships that could have appeared to influence the work reported in this paper.

## Footnotes

1 http://epub.cnipa.gov.cn/Index.

2 https://www.wipo.int/classifications/ipc/green-inventory/home.

3 https://patents.google.com/.
